# Characterization of brown adipose tissue thermogenesis in the naked mole-rat (*Heterocephalus glaber*), a heterothermic mammal

**DOI:** 10.1038/s41598-020-74929-6

**Published:** 2020-11-10

**Authors:** Yuki Oiwa, Kaori Oka, Hironobu Yasui, Kei Higashikawa, Hidemasa Bono, Yoshimi Kawamura, Shingo Miyawaki, Akiyuki Watarai, Takefumi Kikusui, Atsushi Shimizu, Hideyuki Okano, Yuji Kuge, Kazuhiro Kimura, Yuko Okamatsu-Ogura, Kyoko Miura

**Affiliations:** 1 Department of Aging and Longevity Research, Kumamoto University, Kumamoto, 860-0811 Japan; 2 Division of Immunobiology, Institute for Genetic Medicine, Hokkaido University, Hokkaido, 060-0815 Japan; 3 Laboratory of Radiation Biology, Department of Applied Veterinary Sciences, Faculty of Veterinary Medicine, Hokkaido University, Hokkaido, 060-0818 Japan; 4 Central Institute of Isotope Science, Hokkaido University, Hokkaido, 060-0815 Japan; 5 Laboratory of Integrated Molecular Imaging, Department of Biomedical Imaging, Graduate School of Biomedical Science and Engineering, Hokkaido University, Hokkaido, 060-0815 Japan; 6 Program of Biomedical Science, Graduate School of Integrated Sciences for Life, Hiroshima University, 3-10-23 Kagamiyama, Higashi-Hiroshima, Hiroshima, 739-0046 Japan; 7 Laboratory of Veterinary Surgery, Joint Department of Veterinary Medicine, Faculty of Applied Biological Sciences, Gifu University, 1-1 Yanagido, Gifu, 501-1193 Japan; 8 Laboratory of Human-Animal interaction and Reciprocity, School of Veterinary Medicine, Azabu University, Sagamihara, 252-5201 Japan; 9 Laboratory of Behavioral Neuroscience, Institute for Quantitative Biosciences, The University of Tokyo, Tokyo, 113-0032 Japan; 10 Division of Biomedical Information Analysis, Institute for Biomedical Sciences, Iwate Medical University, Shiwa, 028-3694 Japan; 11 Department of Physiology, Keio University School of Medicine, Tokyo, 160-8582 Japan; 12 Laboratory of Biochemistry, Faculty of Veterinary Medicine, Hokkaido University, Hokkaido, 060-0818 Japan; 13 Center for Metabolic Regulation of Healthy Aging, Kumamoto University, Kumamoto, 860-8556 Japan

**Keywords:** Zoology, Animal physiology

## Abstract

The naked mole-rat (NMR) is a heterothermic mammal that forms eusocial colonies consisting of one reproductive female (queen), several reproductive males, and subordinates. Despite their heterothermy, NMRs possess brown adipose tissue (BAT), which generally induces thermogenesis in cold and some non-cold environments. Previous studies suggest that NMR-BAT induces thermogenesis by cold exposure. However, detailed NMR-BAT characteristics and whether NMR-BAT thermogenesis occurs in non-cold environments are unknown. Here, we show beta-3 adrenergic receptor (ADRB3)-dependent thermogenic potential of NMR-BAT, which contributes to thermogenesis in the isolated queen in non-cold environments (30 °C). NMR-BAT expressed several brown adipocyte marker genes and showed noradrenaline-dependent thermogenic activity in vitro and in vivo. Although our ADRB3 inhibition experiments revealed that NMR-BAT thermogenesis slightly delays the decrease in body temperature in a cold environment (20 °C), it was insufficient to prevent the decrease in the body temperatures. Even at 30 °C, NMRs are known to prevent the decrease of and maintain their body temperature by heat-sharing behaviors within the colony. However, isolated NMRs maintained their body temperature at the same level as when they are in the colony. Interestingly, we found that queens, but not subordinates, induce BAT thermogenesis in this condition. Our research provides novel insights into NMR thermoregulation.

## Introduction

Non-shivering thermogenesis in brown adipose tissue (BAT) helps maintain the body temperatures of homeothermic mammals in cold environments^1^. BAT specifically expresses uncoupling protein 1 (UCP1), which dissipates the energy produced from lipid and glucose metabolism as heat, rather than adenosine triphosphate synthesis, by increasing the proton conductance of the inner mitochondrial membrane. BAT has also been shown to be involved in thermogenesis in non-cold environments in response to stimuli, such as diet, social defeat, handling, or the presence of an intruder^2–5^. The detailed mechanisms involved in this process have been intensively explored to develop novel treatments for diabetes and other metabolic diseases because BAT thermogenesis improves lipid and glucose metabolism^6–9^. Interestingly, BAT has also been found in some non-homeothermic, non-hibernating mammals, whose body temperatures decrease in cold environments without undergoing social or behavioral adaptations^10,11^. Previous studies have suggested that the BAT thermogenesis in these species also occurs by the injection of noradrenaline^10^ or in non-cold situation^11^.

**Figure 1:**
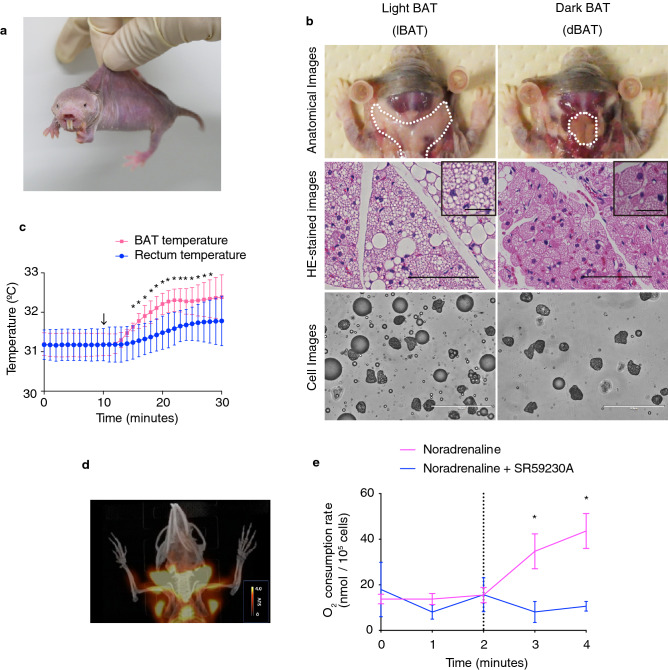
Heterothermic naked mole-rats (NMRs; *Heterocephalus glaber*) possess thermogenic brown adipose tissue (BAT). (**a**) Photograph of an adult NMR. (**b**) Anatomical (top), hematoxylin–eosin (HE)-stained (middle) and cell (bottom) images of light BAT (lBAT) and dark BAT (dBAT). Scale bar = 100 µm for HE-stained images, 25 µm for insets, 200 µm for cell images. (**c**) BAT and rectum temperatures of anesthetized NMRs before and after the *i.p.* injection of 1 mg/kg noradrenaline (arrow) at 30 °C (*n* = 3 animals). *p < 0.05 significantly different from each temperature at 9 min (paired *t*-test). (**d**) Positron emission tomography/computed tomography (PET/CT) imaging of NMR-BAT after the injection of 1 mg/kg noradrenaline and 11 MBq 2-deoxy-2-[^18^F]fluoro-d-glucose ([^18^F]FDG) at 32 °C. (**e**) In vitro oxygen consumption rates of isolated adipocytes after the injection of 1 µM noradrenaline (dotted line) with or without pre-incubation with 10 µM SR59230A (*n* = 3 animals per treatment). *p < 0.05 significantly different from SR59230A treated cells (paired *t*-test). All data are presented as means ± SEM with the exception of (**c**), which are means ± SD.

The naked mole-rat (NMR; *Heterocephalus glaber*; Fig. 1a) is an African heterothermic mammal that is hairless with thin skin and is known as the longest-living rodent in the world, with extraordinary cancer resistance^12–14^. NMRs live in colonies comprising many individuals (average 70–80 individuals^12^) and form complex underground systems of tunnels, which can reach a total length of 3–5 km per colony^15,16^. These tunnels connect chambers that are used for different activities, including nests, toilets, food storage sites, and garbage spots. Interestingly, heterothermic NMRs in a colony can maintain “behaviorally homeothermic” states, regulating their body temperatures by huddling together in the nest. Additionally, they share their heat during passing over and under the other NMRs in tunnels, or decrease their body temperatures by moving to cooler areas within the tunnel network^17,18^. NMRs are also known for their unique eusociality—in a colony of up to 300 individuals, only one female (queen) and one to three males are reproductive, with all other members being sexually immature and working as subordinates^19,20^.

Daly et al. have previously reported that NMRs have BAT in the interscapular region and in the area around the cervix^10^. Hislop et al. and Goldman et al. indicated that NMR-BAT can induce non-shivering thermogenesis based on the increased rate of in vivo oxygen consumption of NMRs after noradrenaline injection^21,22^. Several groups have shown that NMRs are capable of thermoregulation and maintaining body temperatures above the ambient levels in cold conditions^17,23–25^ although NMRs cannot maintain their body temperatures in more severe conditions such as cold environments with cool wind^26^. These studies lend credence to the thermogenic potential of NMR-BAT and BAT-dependent thermogenesis in a cold environment. In contrast, NMR-specific mutations that may contribute to functional inability in UCP1 have been reported^27^. Moreover, no direct measurements of BAT thermogenesis using a thermoprobe have been performed in NMRs. Furthermore, it is still unknown whether NMR-BAT thermogenesis occurs in a non-cold environment. Therefore, we aimed to perform a direct measurement and characterization of NMR-BAT both in vitro and in vivo.

In this study, we investigated the molecular and histological characteristics of NMR-BAT, its thermogenic ability, and the NMR-BAT thermogenesis at 20 °C (a cold temperature for NMRs) and 30 °C (a non-cold temperature for NMRs^26^). We demonstrate that NMRs possess a substantial amount of BAT with thermogenic activity. Although NMR-BAT thermogenesis slightly delays the decrease in body temperature at 20 °C, it was insufficient to prevent the decrease in the body temperatures. Interestingly, NMR queens, but not subordinates, induce BAT thermogenesis when in an isolated at 30 °C. Our results show that the NMR-BAT is indeed thermogenic and induces thermogenesis in physiological, cold and non-cold environments. This study provides new insights into the thermoregulation in this heterothermic rodent.

## Results

### Identification and examination of thermogenic BAT in naked mole-rats

In our laboratory, NMRs are housed in acrylic chambers connected by acrylic tunnels that are maintained at 30 ± 0.5 °C, which represents a non-cold environment for NMRs^26^ (Fig. S1). We first performed a detailed characterization of NMR-BAT based on dissection and histological analysis. We identified two types of BAT around the cervix of NMRs: subcutaneous light BAT (lBAT), located in the interscapular region and around the cervix, and dark BAT (dBAT), located in deep regions under the cervical muscle (Figs. 1b and S2a). Hematoxylin–eosin (HE) staining and the isolation of adipocytes showed that dBAT mostly consisted of multilocular adipocytes, whereas lBAT was comprised of a mixture of multilocular and unilocular adipocytes (Figs. 1b and S2b). Because thermogenically active adipocytes contain smaller lipid droplets than inactive adipocytes^28^, we measured the size of the lipid droplets in the HE-stained images, which showed that dBAT contained significantly smaller lipid droplets than the interscapular BAT of Crl:CD1 (ICR) mice (Fig. S2c). The average percentage of the total BAT per gram body weight was 1.88% (Fig. S2d).

Quantitative reverse transcription-polymerase chain reaction (qRT-PCR) showed that brown adipocyte marker genes reported in humans^29^ and mice^30^, such as *UCP1*, Zic family member 1 (*ZIC1*), peroxisome proliferator-activated receptor gamma coactivator 1 alpha (*PGC1α*), and iodothyronine deiodinase 2 (*DIO2*), were highly expressed in dBAT and lBAT (Fig. S2e). In contrast, for the marker genes of beige adipocyte, an inducible type of thermogenic adipocyte^29,30^, such as T-box transcription factor (*TBX1*) and transcriptional coactivator of the p300/CBP-mediated transcription complex (*CITED1*), were not upregulated in dBAT or lBAT (Fig. S2e). A gene ontology enrichment analysis further showed that processes related to the generation of precursor metabolites and energy, including the monocarboxylic acid metabolic process, acyl-CoA metabolic process, mitochondrial electron transport, electron transport from ubiquinol to cytochrome c, triglyceride metabolic process, response to fatty acid, glucose-6-phosphate metabolic process, and electron transport from cytochrome c to oxygen, were activated in dBAT, indicating that active metabolism occurs in this tissue (Fig. S2f). Furthermore, western blotting showed that the UCP1 protein is highly expressed in dBAT and lBAT (Fig. S3).

To directly evaluate the thermogenic ability of NMR-BAT, we measured the BAT temperature and the rectum temperature following the administration of noradrenaline through a thermoprobe inserted into the BAT and rectum of anesthetized NMRs. We found that the noradrenaline injection caused the BAT temperature to increase by approximately 1.2 °C. We also found that the rectum temperature gradually increased by 0.6 °C after the noradrenaline injection although this increase was statistically insignificant (Fig. 1c). A positron emission tomography/computed tomography (PET/CT) analysis further showed that 2-deoxy-2-[^18^F]fluoro-d-glucose ([^18^F]FDG) was strongly taken up by the BAT of the noradrenaline-injected NMRs, with a clear “neck warmer”-like distribution of [^18^F]FDG around the cervix, in addition to its presence in the interscapular regions (Fig. 1d and Video S1).

To evaluate whether NMR-BAT thermogenesis depends on the beta-3 adrenergic receptor (ADRB3), which plays a critical role in BAT thermogenesis and has a relatively specific expression in the adipose tissues^1^, we measured noradrenaline-induced oxygen consumption rate of brown adipocytes after a noradrenaline treatment in the presence or absence of the ADRB3 inhibitor SR59230A, using adipocytes isolated from a mixture of dBAT and lBAT. We found that the stimulation with noradrenaline caused the rapid increase in the oxygen consumption rate of brown adipocytes, but this increase was not observed with the pretreatment using SR59230A (Fig. 1e).

### Induction of NMR-BAT thermogenesis that slightly delays the decrease in body temperature in a cold environment

Next, we investigated the roles of NMR-BAT in physiological conditions. Because NMR skin is almost hairless and quite thin (Fig. 1a), BAT thermogenesis can be monitored by measuring the cervix surface temperature with a thermal camera. To determine the effective distance between our thermal camera and NMRs in order to measure their body temperatures (approximately 32 °C), we measured the temperature of a thermostable object (30–34 °C) at various distances. We found that the temperatures measured by the thermal camera did not differ significantly within a range of 1 m (Fig. S4a,b). Therefore, the measurements of NMRs were performed within a 50 cm distance in our experiments. We found that the cervix surface temperature was correlated with the BAT temperature, as measured by the thermoprobe (Fig. S4c).

**Figure 2:**
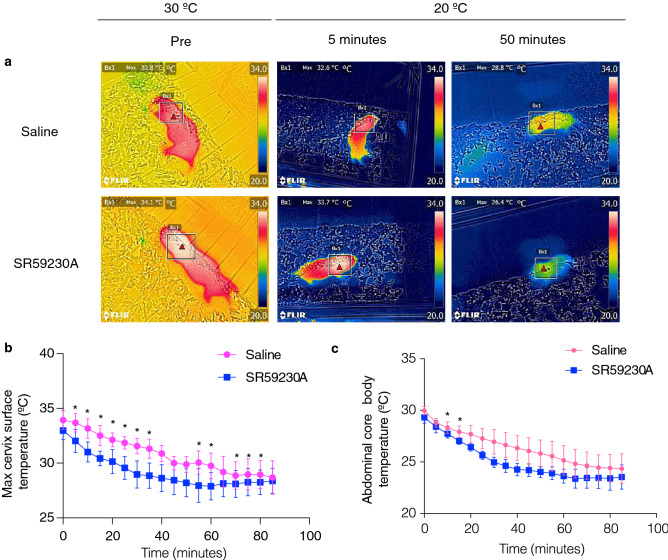
Brown adipose tissue (BAT) thermogenesis in naked mole-rat (NMR; *Heterocephalus glaber*) subordinates after cold exposure. (**a**) Thermal images of the declining body surface temperatures of NMRs during cold exposure (20 °C) following the injection of saline or 20 mg/kg SR59230A (*n* = 4 animals per treatment). (**b**) Maximum cervix surface temperatures monitored by a thermal camera (*n* = 4 animals) and (**c**) abdominal core body temperatures recorded by a telemetry probe inserted into the abdominal cavity (*n* = 3 animals) of NMRs during cold exposure (20 °C) following the injection of saline or 20 mg/kg SR59230A. *p < 0.05 significantly different from SR59230A treated sample (paired *t*-test).

To evaluate the thermogenic ability of BAT in a cold environment for NMRs (20 °C), we monitored the cervix surface temperature and the abdominal core body temperature of free-moving NMR subordinates using a thermal camera and a temperature telemetry system simultaneously. We measured the cervix temperatures of each NMR in the isolated situation because NMRs are known to enter a behaviorally homeothermic state by engaging in a heat-sharing behavior among others in their colony^17,18^. When NMRs were isolated from the colony and transferred to a room at 20 °C, the body and cervix surface temperatures gradually reduced. We found that SR59230A initially accelerated the drops in both temperatures; however, SR59230A did not induce further decrease at equilibrium (Fig. 2a–c). These results suggest that although BAT thermogenesis contributed to a delay in the decrease in body temperature after cold exposure, it was insufficient to prevent the decrease in the body temperature at 20 °C.

### Induction of BAT thermogenesis in naked mole-rat queens under isolated, non-cold conditions

**Figure 3:**
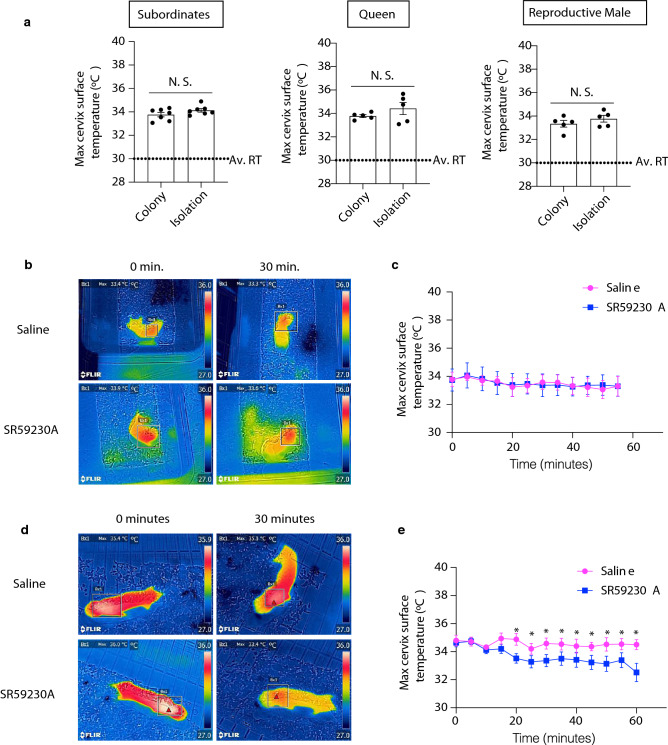
Isolated naked mole-rat (NMR; *Heterocephalus glaber*) queen exhibits BAT thermogenesis. (**a**) Maximum cervix surface temperatures of NMRs staying together in the colony (colony) or isolated from the colony (isolation). In the colony data, each point represents the average temperatures of individual NMRs measured at six times in the colony (three times in the nest and three times outside the nest). In the isolation data, each point represents the average temperatures of individual NMRs recorded every 30 min over 8 h. *n* = 5 animals for the queen and reproductive male, *n* = 7 animals for the subordinate. Av. RT, the average room temperature, (**b**) Representative images and (**c**) time course of changes in the maximum cervix surface temperatures of socially isolated subordinates following the *i.p.* injection of saline or 20 mg/kg SR59230A (*n* = 5 animals). Measurement began after the cervix surface temperature became stable. *p < 0.05 significantly different from SR59230A treated animals (paired *t*-test). Data are presented as means ± SEM. (**d**) Representative images, and (**e**) time course of changes in the maximum cervix surface temperatures of socially isolated queens, following the *i.p.* injection of saline or 20 mg/kg SR59230A (*n* = 5 animals); *p < 0.05 significantly different from SR59230A treated animals (paired *t*-test). Data are presented as means ± SEM.

We found that BAT thermogenesis had a slight but insufficient effect on supporting the body temperature of NMRs in a cold environment (20 °C) (Fig. 2). Therefore, we hypothesized that NMR-BAT may also play a role in non-cold environments (30 °C). Interestingly, we found that NMRs isolated from their colony did not decrease their body temperatures (Figs. 3a and S5a). To test whether the thermogenesis of the isolated subordinates depended on BAT, we injected NMRs with SR59230A and measured the body temperature of individuals after isolation by the thermal camera. However, no significant change was observed in the subordinates (Fig. 3b,c).

Next, we tested whether the BAT thermogenesis occurred in other social status of NMRs. We injected SR59230A into an isolated queen and measured the body temperature of the individuals. As a result, we found that the body temperatures of the socially isolated queens significantly decreased following the SR59230A injection (Fig. 3d,e). On the other hand, we did not find a significant difference in the oxygen consumption rates between the queens and the other members in isolation (Fig. S5b). A previous study reported that the body weight and age were higher in the queen than in the subordinates^27^; therefore, we evaluated the relationships between the cervical body surface temperatures and the body weight and ages in the isolated subordinate NMRs. We found that weight was not significantly correlated (Fig. S5c), and age was negatively correlated with the body temperatures of the isolated subordinate NMRs (Fig. S5d). Additionally, we did not find significant differences in body temperature between the sexes (Fig. S5e).

## Discussion

In this study, we provided direct evidence in support of the thermogenic potential of BAT in heterothermic NMRs, which is ADRB3 dependent. We show that BAT thermogenesis was insufficient to prevent the decrease in the body temperatures of the NMRs in 20 °C although it can slightly delay the decrease in the body temperatures of NMRs. Furthermore, in 30 °C, BAT thermogenesis contributes to maintaining the body temperature of the isolated queen. This research provides in vitro and in vivo evidence of NMR-BAT thermogenesis in physiological conditions that have not previously been studied.

A previous study suggested that the NMR *UCP1* gene has a unique sequence that may contribute to the inability of thermogenesis in NMRs^27^; however, our results clearly show that NMR-BAT has a thermogenic potential. Compared to the previous data on the in vitro thermogenic ability of mouse brown adipocytes, measured by the same equipment^31^, the thermogenic potentials of NMR brown adipocytes do not seem less than those of mice. Of course, we did not compare the levels of thermogenic ability of NMR brown adipocyte to that of mice at the same time; therefore, further investigation is required to determine whether the thermogenic potential of NMR brown adipocytes is equal to that of other species.

Although our results clearly showed the thermogenic ability of NMR-BAT, the body temperature was decreased to about 28 °C (cervical temperature) and 23.5 °C (abdominal temperature) at 20 °C. This result may reflect the fact that NMRs have high heat dissipation because of their small body size and thin and hairless skin^25^. Importantly, even with the ADRB3 inhibitor, the body temperature of NMRs was higher than the ambient temperature. We observed that NMRs were shivering at 20 °C; thus, the shivering thermogenesis, along with BAT-dependent thermogenesis, may have contributed to the maintenance of the body temperature at 20 °C. Although we used the maximum volume of SR59230A that was recommended in the animal experiment guideline^32^, we could not exclude the possibility that the non-shivering thermogenesis was not fully inhibited by SR59230A.

In our experiment, humidity at 20 °C (45–55%) was lower than that at 30 °C (69–80%) (Table S2), and this difference may have influenced the evaporative water loss that contributes to the decrease in the NMR body temperatures. Considering the body temperatures higher than the ambient temperatures even in this cold condition, our data supports previous studies that mentioned the thermogenic potentials of NMRs in a cold environment^17,23,24^.

It is of note that the body surface cervical temperatures were higher than abdominal temperatures when measured by the thermal camera with or without ADRB3 inhibitor. In general, the cervical region has major blood vessels, and in NMRs, the skin is thin and the amount of adipocyte in the skin is low^33^. These NMR features may have contributed to the higher cervical temperature. In the future, measurement of the difference in temperatures between the brain and other organs may reveal the unique features of thermoregulation in NMRs.

We observed that only queens induce BAT thermogenesis when subjected to an isolated situation at 30 °C. Previous reports on mice and rats have indicated that chronic social isolation affects the metabolism and volume of adipose tissues, including BAT^34,35^, and that psychological stresses, such as social defeat^3^, handling^4^, or the presence of an intruder^5^, have been shown to induce BAT thermogenesis. Recent studies have revealed that cortisol concentration is also upregulated in NMRs after social isolation^36,37^, suggesting that social isolation induces psychological stress in NMRs. Importantly, according to the previous studies^38,39^, we did not use lactating queens in the experiments of Fig. 3d,e. Also, one queen may be pregnant in the experiments of Fig. 3d,e., but at a very early stage, which would have a small impact on the interpretation of the results (Table S3). The differences in stress levels between the queen and other colony members and the neurological systems to transmit the stress to BAT during social isolation in NMRs, which are still unknown, may have contribute to the observed difference in BAT thermogenesis in the isolated situation. Another possibility is that the difference in the BAT thermogenesis between the queen and the other members may result from differences in the thermogenic function or volume of BAT. However, we were unable to collect and examine BAT in the queen by dissection due to the limited number of queens in our laboratory. Therefore, further experiments are required to investigate the mechanism underlying this queen-specific activation of BAT under isolation in a non-cold environment.

Moreover, our observations suggest the possibility that the queen might be more resistant to decreasing body temperature in a cold environment than other members. However, we could not measure the core body temperature of the queen in a cold environment by inserting the thermoprobe into the abdominal cavity because this would have been too invasive, and the number of reproductive queens was quite limited. Elucidating this factor will be another important issue for future work. Additionally, the oxygen consumption rate of the isolated queen was not significantly higher than that of the other colony members although BAT thermogenesis in the isolated queen did occur. This result suggests that differences in the metabolism of organs other than BAT between the queen and the other members of the colony should be explored further in future research.

In isolated subordinates, we observed higher body temperatures than the ambient temperatures in 30 °C; however, the ADRB3 inhibitor did not decrease the body temperature. Although we cannot deny the possibility that more sensitive measurements can provide different results, our results may suggest that the isolated subordinates increase their body temperatures by BAT-independent mechanisms such as activity-dependent thermogenesis.

A current open question remains whether NMRs staying together in the colony also induce BAT thermogenesis. However, NMRs display the heat-sharing behavior only when inside the colony, and the intraperitoneal injection of SR59230A is not suitable for suppressing BAT thermogenesis for a long time period. Although we tried to irreversibly suppress BAT thermogenesis by cutting the sympathetic nerve projecting into the BAT, we failed because this method was too invasive. Developing methods that suppress BAT thermogenesis for long periods such as using the miniature osmotic pump^40^ and allowing for more sensitive measurement of BAT temperatures will contribute to the further understanding of the function and role of NMR-BAT.

In conclusion, we revealed that the heterothermic NMR-BAT is thermogenic, inducing thermogenesis in physiological, cold, and non-cold environments. This work provides novel insights into the previously unclear role of BAT in this heterothermic mammal. Further studies of BAT thermogenesis in the NMR and other non-homeothermic animals should continue to advance our understanding of the unexpected roles of BAT in animal homeostasis.

## Methods

### Study organisms

The NMRs used in this study were maintained at Kumamoto University and Hokkaido University where they were housed in four to 10 acrylic chambers that were connected by acrylic tunnels, at 30 °C ± 0.5 °C and 55% ± 5% humidity with a 12 h light/12 h dark cycle. The effect of social isolation was evaluated using 0.73- to 14.8-year-old subordinates (mean; 4.82, S.D.; 4.59), 3.6- to 14.5-year-old queens (mean; 9.30, S.D.; 4.75), and 4.4- to 11.9-year-old reproductive males (mean; 8.46, S.D.; 3.07) (Fig. 3a). The oxygen consumption rates were assessed using 0.85- to 3.24-year-old subordinates (mean; 2.01, S.D.; 0.86), 2.43- to 7.77-year-old queens (mean; 5.53, S.D.; 2.27), and 3.3- to 10.3-year-old reproductive males (mean; 7.81, S.D.; 1.66) (Fig. S5b). The effect of SR59230A on isolated NMRs was evaluated using 1.4- to 1.7-year-old subordinates (mean; 1.54, S.D.; 0.14) and 2.6- to 13.5-year-old queens (mean; 6.88, S.D.; 4.35) (Fig. 3b–e). The body temperatures of the isolated NMRs were evaluated for comparison according to age, body weights, and sex were assessed using 0.81- to 13.6-year-old subordinates (mean; 4.25, S.D.; 4.01) that weights were 21.15 to 51.79 g (mean; 34.7, S.D.; 7.12) (Fig. S5c–e). JcI:ICR mice were purchased from CLEA Japan, Inc., and adipose tissues were collected from 1- to 2-year-old subordinates and 6-week-old mice for cytological and histological analyses. In our experiment, we selected queens for the experiments with two constraints; (1) the colony with the queen had no pups in the lactating period, (2) the queen did not appear to be pregnant. After the experiment, we checked the queen giving birth records before and after the experiments (Table S3).

All experimental procedures were permitted by the Institutional Animal Care and Usage Committees of Kumamoto University (Approval No. A30-043) and Hokkaido University (Approval No. 14-0065). Our experiments were conducted according to the guidelines of the Institutional Animal Care and Usage Committees of Kumamoto University and Hokkaido University, which were in accordance with the Guide for the Care and Use of Laboratory Animals (United States National Institutes of Health, Bethesda, MD).

### HE staining and measurement of the lipid droplet size

The NMR and mouse adipose tissues were fixed with 4% paraformaldehyde in phosphate-buffered saline at pH 7.4. HE staining was performed by Sapporo General Pathology Laboratory Co., Ltd. (Hokkaido, Japan), and images of the HE-stained samples were acquired with a BZ-X 710 fluorescence microscope (KEYENCE). The lipid droplet size was measured using a BZ-X image analyzer (KEYENCE).

### mRNA-sequencing analysis

RNA was extracted from dBAT and inguinal white adipose tissue (iWAT) using Trizol reagent (Life Technologies) in accordance with the manufacturer’s protocol. A Qiagen RNeasy column was used for further purification, and genomic DNA was excluded using the TURBO DNA-free kit (Invitrogen). RNA quantity and quality were measured by Qubit (Invitrogen) and a 2100 Bioanalyzer using the RNA 6000 Nano Kit (Agilent Technologies). The TruSeq RNA Library Prep Kit v2 (Illumina) was used for library preparation in accordance with the manufacturer’s protocol. The acquired library was quantified using the High Sensitivity DNA Kit (Agilent Technologies) and Kapa Library Quantification Kit (Kapa Biosystems) with the Applied Biosystems ViiA7 Real-Time PCR System (Applied Biosystems) using the manufacturer’s protocol. The library was loaded into a flow cell for cluster generation with the TruSeq Rapid SR Cluster Kit (Illumina) and was sequenced using the Illumina Hiseq 2500 System to obtain single-end 100-nucleotide sequences.

The NMR reference genome (HetGla_female_1.0) and annotation files downloaded from Ensembl 92 (https://www.ensembl.org) were used for data analysis. The acquired fastq files were trimmed using Trim Galore ver. 0.4.4.^41^, and the transcriptional abundance (transcripts per million [TPM]) in the trimmed fastq files was calculated using RSEM ver. 1.2.31 with Bowtie 2^42,43^.

For the gene ontology enrichment analysis, the calculated TPM of dBAT was compared with that of iWAT, and the top 200 upregulated genes in dBAT were analyzed by Metascape using gene ontology annotation of the mouse^44,45^.

### qRT-PCR

Total RNA was extracted from NMR adipose tissues using the RNeasy Lipid Tissue Mini Kit (Qiagen), and genomic DNA was eliminated using the TURBO DNA-free Kit (Invitrogen) following the manufacturers’ protocols. Reverse transcription reactions were carried out using ReverTra Ace qPCR RT Master Mix (TOYOBO) with 300 ng of RNA as a template. The resulting cDNA was prepared for qPCR using Thunderbird qPCR Mix (TOYOBO) in a 384-well plate with the primers listed in Table S1. qPCR was performed on a CFX384 Touch Real-Time PCR Detection System (Bio-Rad).

### In vivo measurement of BAT thermogenesis

NMRs were anesthetized with 0.1 µg/g medetomidine hydrochloride (Dorbene Vet; Kyoritsu Seiyaku Co.), 4 µg/g midazolam (Dormicum; Asteras Pharma Inc.), and 5 µg/g butorphanol (Vetorphale; Meiji Seika Pharma Co.). BAT temperature and rectum temperature were simultaneously measured using a thermoprobe (plastic-coated thermistor, 1 mm diameter). To measure the BAT temperature, the skin above the interscapular region was incised without injury to the vasculature and nerves, and the thermoprobe was inserted under the lBAT. A thermoprobe was also inserted into the rectum at the same time. Once the BAT and rectum temperatures stabilized, noradrenaline was injected into the abdominal cavity, and the temperatures of the BAT and rectum were recorded over 30 min. Based on a previous study, we used 1 mg/kg of noradrenaline in our experiment^21^. All procedures were performed in a non-cold environment (30 °C ± 0.5 °C), and the NMRs were placed on a hot plate at 32 °C (NHP-M30N; NISSIN RIKA) during the experiment.

### In vivo measurement of oxygen consumption

The in vivo oxygen consumption rate was measured using an O_2_/CO_2_ metabolism-measuring system (MK-5000RQ6; Muromachi Kikai) and MMS-ML/6 software (Muromachi Kikai). MK-5000RQ6, using a paramagnetic oxygen sensor with a sensitivity of 0.05%/h, measured the oxygen concentration by pulling air from the chamber with the correction by the partial pressure of water vapor. Gas flow rates were 0.35–0.40 L/min, which were determined based on the CO_2_ concentration in the chamber. The analyzed sequence duration was 1 min, and the measurement of oxygen in the chamber or in air were performed every 3 min. The oxygen sensor was calibrated by the calibrated gas every 1 h. The oxygen consumption of a single NMR was measured at 30 °C ± 0.5 °C in a sealed chamber (300-mm width, 190-mm length, 250-mm height). The NMRs were habituated to the sealed chamber for 2 h before testing, and the oxygen consumption rate was recorded for 4 h during the daytime.

### In vitro measurement of adipocyte oxygen consumption

NMR brown adipocytes were isolated from lBAT and dBAT as previously described^46^. Briefly, incised NMR adipose tissues were incubated in Krebs–Ringer bicarbonate-HEPES (KRBH) buffer (130 mM Na^+^, 4 mM K^+^, 0.75 mM Ca^2+^, 1 mM Mg^2+^, 121.5 mM Cl^−^, 10 mM HCO_3_^−^, 1 mM Mg^2+^, 4 mM HPO_4_^2−^, 30 mM HEPES, and pH 7.4) with 1% fatty acid-free bovine serum albumin (Wako), 6 mM glucose, and 1 mg/mL collagenase (Sigma) at 37 °C for 1 h, with shaking at 90 rpm/min. After filtering the suspension through a 200 µM nylon filter, the filtrate was centrifuged at 50×*g* for 2 min. The floating adipocytes were then collected and suspended with measurement buffer (KRBH buffer containing 4% bovine serum albumin and 2.7 mM glucose) and then recentrifuged and washed three times with the measurement buffer. The acquired adipocytes were incubated at room temperature for 1 h before measurement.

The oxygen consumption rate was measured using a Clark-style oxygen electrode in a water-jacketed Perspex chamber at 37 °C with the StrathKelvin 782 2-Channel Oxygen System (StrathKelvin Instruments). The chamber volume was 1 ml. The diluted adipocytes (1/10–1/20 volume) were added to the chamber along with the measurement buffer. Once a stable oxygen consumption rate was recorded, noradrenaline at a final concentration of 1 µM was injected into the chamber^31^. To examine the effect of the noradrenaline receptor inhibition, 10 µM of SR59230A (Sigma) was injected 10 min before injecting noradrenaline^47^. After the measurement, the brown adipocytes were counted using a hemocytometer.

### Measurement of the body temperature via a thermal camera and telemetry probe at 20 °C

Body surface temperatures were monitored using a thermal camera (CPA-E6A; FLIR). The range, thermal sensitivity, and precision of this camera are − 20 to 250 °C, 0.06 °C, and ± 2 °C, respectively. The acquired data were analyzed by FLIR Tools ver. 2.1 (FLIR) based on the following parameters: distance of 1 m, emissivity of 0.98^48^, and the reflected temperature sets to ambient temperature. The lid of the chamber was removed when the body surface temperatures were measured. The abdominal core body temperature was measured using a telemetric probe (G2 E-mitter; STARR Life Sciences Corp.) and an ER4000 receiver (STARR Life Sciences Corp.). The G2 E-mitter was inserted into the abdominal cavity of anesthetized NMRs, and the animals were left for at least 7 days before being used in the experiment. The acquired data were analyzed by VitalView ver. 4.1 (STARR Life Sciences Corp.). For the measurement of the cervical temperature and abdominal core temperature in a cold environment (20 °C), we first injected saline or 20 mg/kg SR59230A to the NMR abdominal cavity in a non-cold environment (30 °C). Then, we moved the NMR in the acrylic cage to a cold environment (20 °C) and measured changes in the cervical temperature and the abdominal core temperature every 5 min for 90 min.

### Measurement of the body temperature via a thermal camera at 30 °C

The body surface temperature of each individual in the colony was measured three times each at inside and outside the nest, and the average of these temperatures was shown as the body temperature in the colony. For the measurement of the cervical temperature of the socially isolated NMRs, we measured the change in the cervical temperature via a thermal camera every 30 min for 8 h after 30 min of social isolation (*n* = 5 animals for the queen and reproductive male, *n* = 7 animals for the subordinate) (we performed a pre-experiment for this assay, measuring the body surface temperatures after isolation every 5 min for 30 min. In this pre-experiment, the cervical temperatures of most NMRs became stable after 30 min). Individual NMRs were moved from the colony to the acrylic chamber at 30 °C ± 0.5 °C and 55% ± 5% humidity. For the measurement of the body surface temperature in socially isolated NMR to compare against the body weight, age, or sex, we measured the change in the cervical temperature via a thermal camera after 30 min of social isolation.

To measure the cervical temperature of the socially isolated NMR with the injection of saline or 20 mg/kg SR59230A to the abdominal cavity, we measured the change in the cervical temperature using the thermal camera every 5 min for 90 min in a non-cold environment (30 ºC) after 30 min of social isolation and the injection of saline or SR59230A. Treatment with either saline or SR59230A was randomized, and the assays were repeated on the same individual.

To validate the accuracy of our thermal camera measurements, we measured the temperature of thermostable objects (Thermopack, Sugiyama-Gen) at various distances, with simultaneous measurement recorded by a mercury thermometer.

### PET/CT imaging

For PET/CT imaging, NMRs were fasted overnight and kept at 30 ± 0.5 °C and 60% humidity. The NMRs were administered 1 mg/kg noradrenaline, following which 11 MBq [^18^F]FDG was injected into the abdominal cavity. PET/CT images were then acquired 1 h after [^18^F]FDG administration using the Inveon small-animal multimodality PET/CT system (Siemens Medical Solutions). PET scanning was performed for 10 min followed by CT scan. During this experiment, the NMRs were maintained under isoflurane anesthesia and were kept at 32 °C. Acquired PET images were reconstructed using the filtered backprojection algorithm with the ramp filter cut-off at the Nyquist frequency. This PET scanner, which consists of 1.5 × 1.5 × 10 mm lutetium oxyorthosilicate crystal elements with a ring diameter of 16.1 cm, yields an effective transaxial field of view (FOV) of 10 cm and an axial FOV of 12.7 cm^49^. The image matrix was 256 × 256 × 159 mm, resulting in a voxel size of 0.388 × 0.388 × 0.796 mm.

### Western blotting

Each adipose tissue was dissected, lysed in the buffer (125 mM Tris–HCl, pH 6.8; 4% SDS and 10% sucrose), and boiled for 10 min. After centrifuging, the supernatant was collected. The protein concentration was measured by TaKaRa BCA Protein Assay Kit (Takara Bio) in accordance with the manufacturer’s protocol. The 15 µg proteins were subjected to SDS-polyacrylamide gel electrophoresis, and then proteins were transferred to the polyvinylidene fluoride membrane. Blocking was performed with 0.5% skim milk for 1 h at room temperature. Blotted membranes were incubated with the primary antibody overnight at 4 °C and the secondary antibody for 1 h at room temperature. We used anti-UCP-1 antibody (Sigma, U6382; 1:1000) and HRP-conjugated anti-rabbit IgG secondary antibodies (CST, #7074; 1:1000). The membrane was visualized by using Amersham ECL Prime Western Blotting Detection Reagent (GE Healthcare) and ImageQuant LAS 4000 Mini (GE Healthcare).

The detected membrane was washed by tris-buffered saline with Tween-20 for 10 min. Then, the membrane was incubated with Ponceau-S staining solution (Beacle, Inc) for 15 min at room temperature. After discarding the Ponceau-S staining solution, the membrane was incubated with 0.1% acetic acid for 2 min at room temperature. Then, the membrane was dried and photographed with a digital camera (COOLPIX S8200, Nikon).

### Measurement of temperature and relative humidity in NMR colony chamber and in isolation chamber

The temperature in the NMR colony chamber and in the isolation chamber was measured by the mercury thermometer, and relative humidity was measured using a humidity monitor (ThermoPro TP-65 Indoor Outdoor Temperature and Humidity Monitor, ThermoPro).

### Statistical analysis

GraphPad Prism (GraphPad) was used for statistical analysis. Data were analyzed using one-way analysis of variance followed by Tukey’s multiple comparison test with a single pooled variance for multiple comparisons (in Fig. S5a,b) or by Dunnet’s multiple comparison test with a single pooled variance (in Figs. S2e, S4a,b). Two groups were compared using an unpaired *t*-test (in Figs. 3a, S2c and S5e) or paired t-test (in Figs. 1c,e, 2b,c, 3c,e). Simple linear regression analysis was performed, as shown in Figs. S4c, S5c,d. All values are presented as mean ± SD or mean ± SEM, as noted.

## Supplementary information

Supplementary Information.

Supplementary Video S1.

## Data Availability

RNA-seq data have been deposited in the DNA Data Bank of Japan database under accession code: DRA007737. Gene expression abundance data have also been deposited in the Genomic Expression Archive under accession code: E-GEAD-294.
