# Supplementary material for: Characterization of brown adipose tissue thermogenesis in the naked mole-rat (Heterocephalus glaber), a heterothermic mammal
Source: Sci Rep. 2020 Nov 10;10:19488. doi: 10.1038/s41598-020-74929-6 (PMC7656259; doi:10.1038/s41598-020-74929-6)
Supplement: Supplementary file 1 — Supplementary Information. [file 41598_2020_74929_MOESM1_ESM.pdf]

Supplementary Material

Manuscript titles

Characterization of brown adipose tissue thermogenesis in the naked mole-rat (*Heterocephalus glaber*), a heterothermic mammal

Authors

Yuki Oiwa, Kaori Oka, Hironobu Yasui, Kei Higashikawa, Hidemasa Bono, Yoshimi Kawamura, Shingo Miyawaki, Akiyuki Watarai, Takefumi Kikusui, Atsushi Shimizu, Hideyuki Okano, Yuji Kuge, Kazuhiro Kimura, Yuko Okamatsu-Ogura and Kyoko Miura

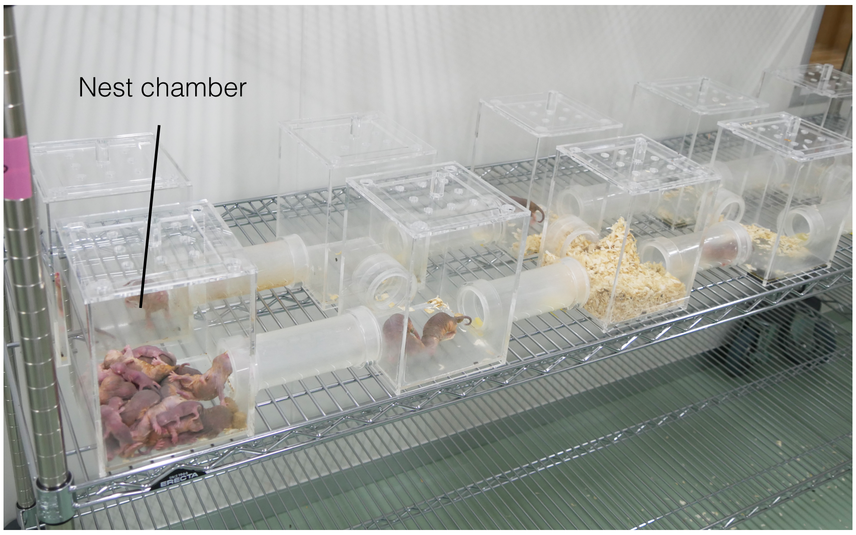
Nest chamber

Figure S1. Images of acrylic chambers connected by acrylic tunnels used to house the naked mole-rats (NMRs; *Heterocephalus glaber*) in the laboratory

The NMRs were housed in acrylic chambers maintained at 30 ± 0.5°C, each of which was assigned a different use. In the nest chamber, NMRs shared heat and became warm by huddling together.

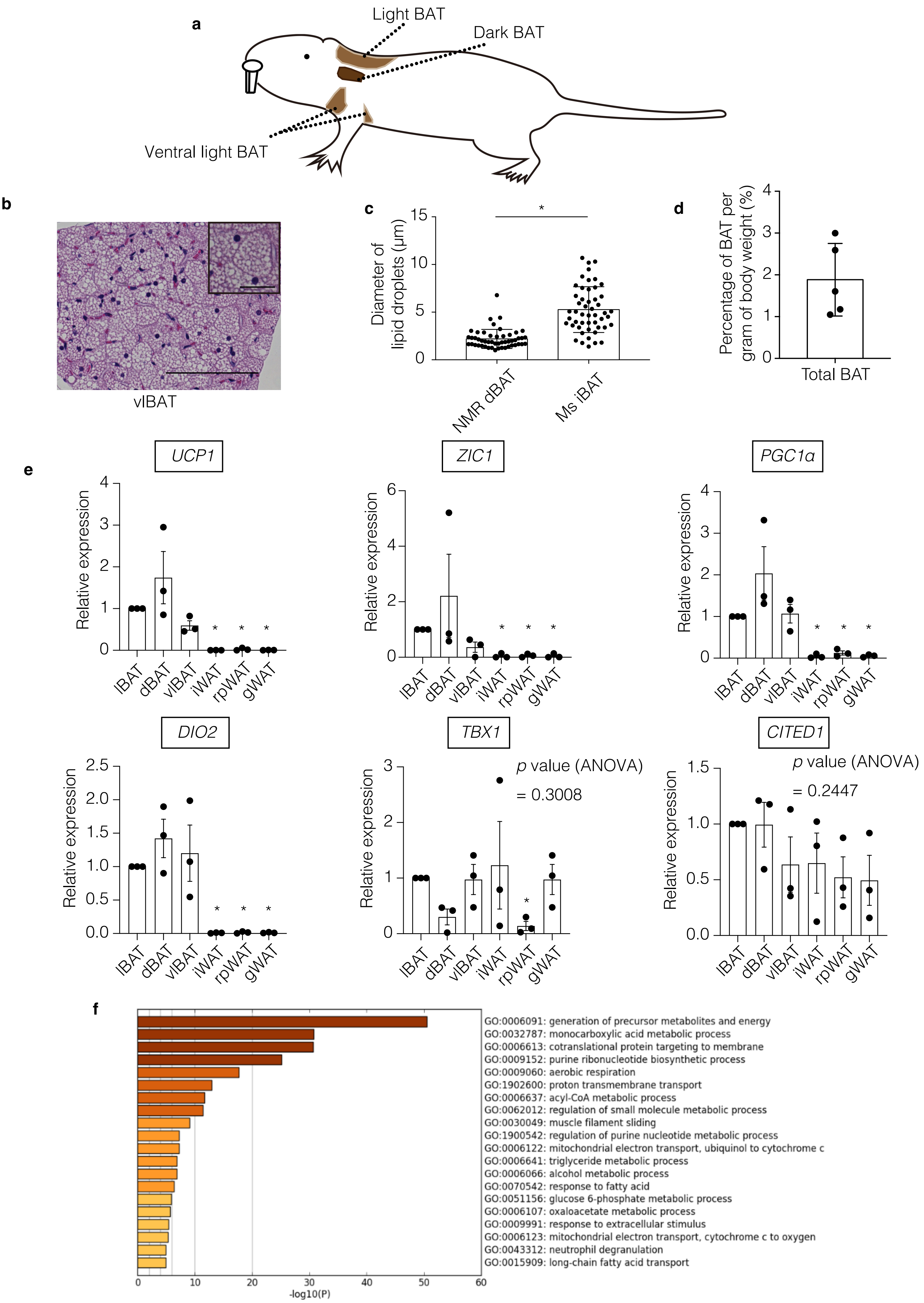
a
Light BAT
Dark BAT
Ventral light BAT
b
vlBAT
c
Diameter of lipid droplets (μm)
NMR dBAT
Ms iBAT
d
Percentage of BAT per gram of body weight (%)
Total BAT
e
UCP1
ZIC1
PGC1α
DIO2
TBX1
CITED1
Relative expression
lBAT
dBAT
vlBAT
iWAT
rpWAT
gWAT
p value (ANOVA) = 0.3008
p value (ANOVA) = 0.2447
f
GO:0006091: generation of precursor metabolites and energy
GO:0032787: monocarboxylic acid metabolic process
GO:0006613: cotranslational protein targeting to membrane
GO:0009152: purine ribonucleotide biosynthetic process
GO:0009060: aerobic respiration
GO:1902600: proton transmembrane transport
GO:0006637: acyl-CoA metabolic process
GO:0062012: regulation of small molecule metabolic process
GO:0030049: muscle filament sliding
GO:1900542: regulation of purine nucleotide metabolic process
GO:0006122: mitochondrial electron transport, ubiquinol to cytochrome c
GO:0006641: triglyceride metabolic process
GO:0006066: alcohol metabolic process
GO:0070542: response to fatty acid
GO:0051156: glucose 6-phosphate metabolic process
GO:0006107: oxaloacetate metabolic process
GO:0009991: response to extracellular stimulus
GO:0006123: mitochondrial electron transport, cytochrome c to oxygen
GO:0043312: neutrophil degranulation
GO:0015909: long-chain fatty acid transport
-log10(P)

Figure S2. Characterization of naked mole-rat (NMR; *Heterocephalus glaber*) brown adipose tissue (BAT)

(a) Schematic diagram showing the location of BAT in NMRs. **(b)** Hematoxylin-eosin (HE)-stained images of ventral light BAT (vlBAT). Scale bar = 100 μm for the HE-stained image, 25 μm for inset. **(c)** Lipid droplet size in NMR dBAT and mouse-interscapular BAT (Ms iBAT). A total of 50 droplets were measured from three different fields of view per tissue type. The data were analyzed using an unpaired *t*-test. **(d)** Percentage of total BAT (sum of light BAT [lBAT], dark BAT [dBAT], and vlBAT) per gram body weight. The data are presented as means ± SD (n = 5 animals). **(e)** Relative expression levels of brown or beige adipocyte marker genes reported in mouse and human in NMR adipose tissues. Expression levels were quantified by quantitative polymerase chain reaction (qPCR) using the primers listed in Table S1 and were normalized to beta-actin (*ACTB*) and the value of lBAT (n = 3 animals). ingWAT, inguinal white adipose tissue (WAT); gWAT, gonadal WAT; rpWAT, retroperitoneal WAT. The data are presented as means ± SEM and were analyzed using one-way analysis of variance followed by Dunnett's multiple comparison test with a single

pooled variance (* $p < 0.05$ significantly different from IBAT). **(f)** Gene Ontology enrichment analysis of the top 200 upregulated genes in the dBAT. The calculated TPM of dBAT was compared with that of iWAT, and the top 200 upregulated genes in dBAT were analyzed by Metascape using gene ontology annotation of the mouse.

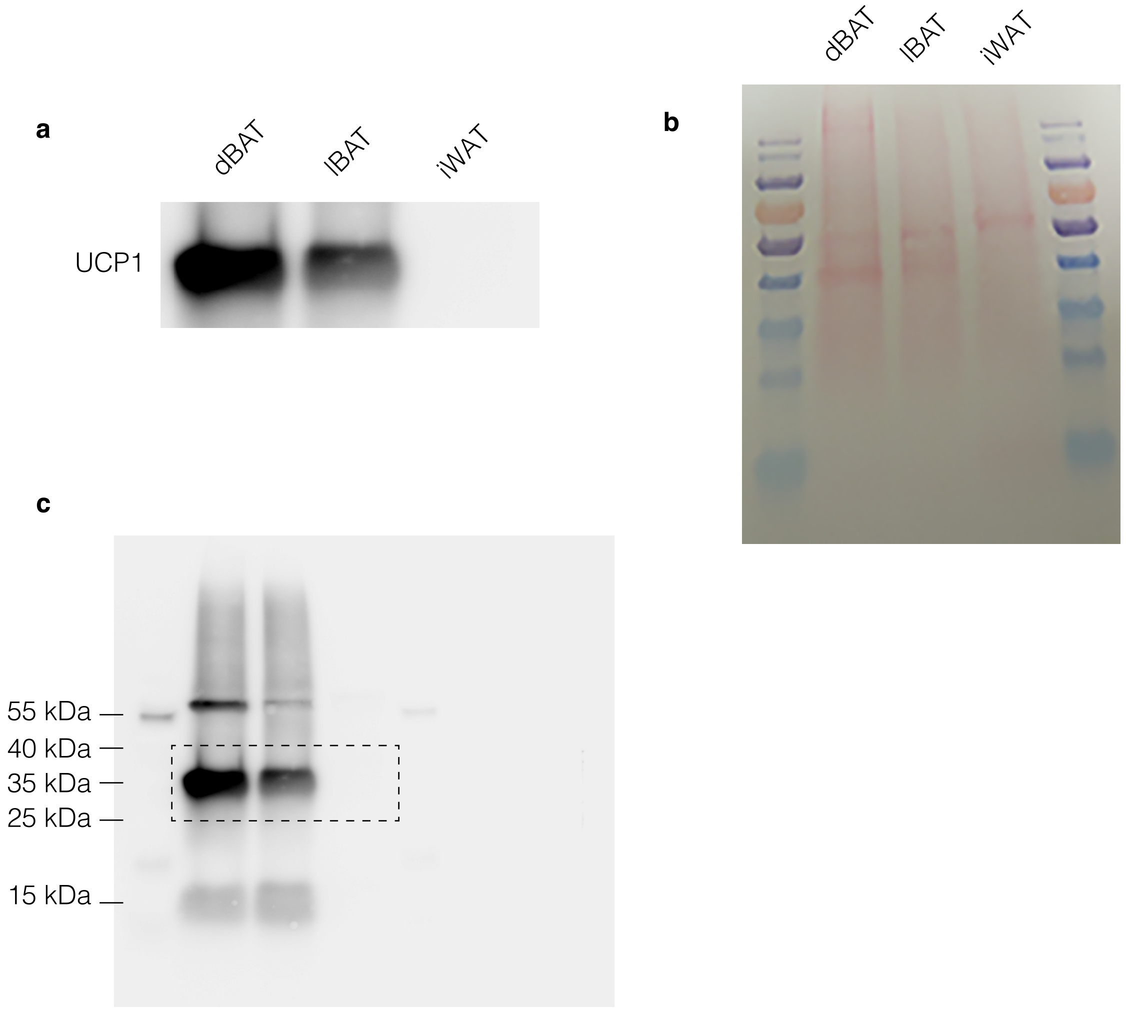
a
dBAT
lBAT
iWAT
UCP1
b
dBAT
lBAT
iWAT
c
55 kDa
40 kDa
35 kDa
25 kDa
15 kDa

Figure S3. Uncoupling protein 1 (UCP1) expression in the naked mole-rat (NMR; *Heterocephalus glaber*)

(a) UCP1 expression was evaluated by western blotting in NMR dBAT, NMR IBAT, and NMR iWAT. **(b)** Ponceau-S staining was performed for the membrane used in (a) after the UCP1 detection. **(c)** Uncropped scanned results of western blot (a).

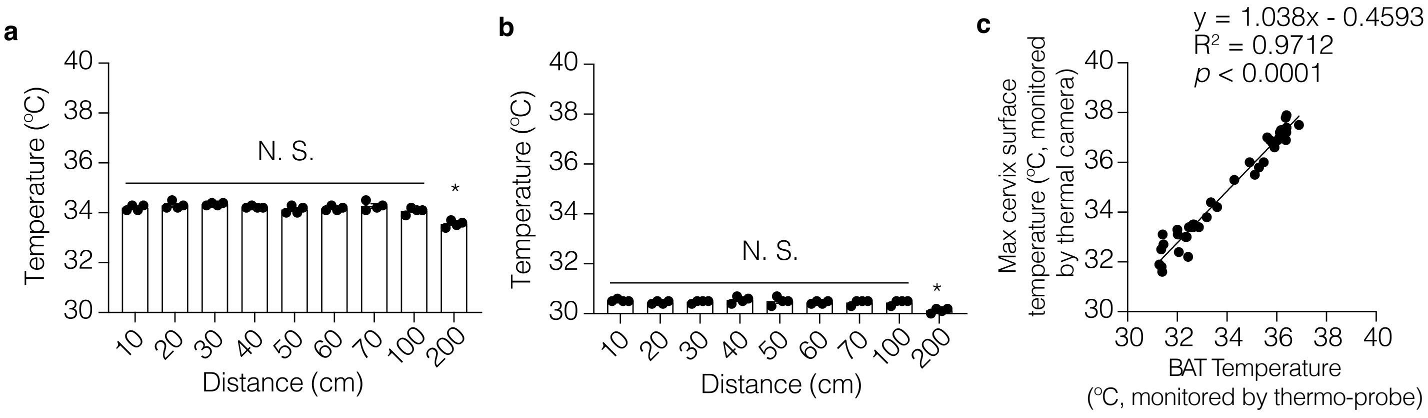

a
b
c
Temperature (ºC)
N. S.
*
10 20 30 40 50 60 70 100 200
Distance (cm)
40 38 36 34 32 30
y = 1.038x - 0.4593
R² = 0.9712
p < 0.0001
Max cervix surface temperature (ºC, monitored by thermal camera)
BAT Temperature
(ºC, monitored by thermo-probe)
30 32 34 36 38 40

Figure S4. Correlation between brown adipose tissue (BAT) temperatures monitored with a thermoprobe and cervix surface temperatures monitored with a thermal camera

(a, b) Temperatures of the thermostable objects (**a;** 34.6–34.9°C, **b;** 31°C, measured by a mercury thermometer) were monitored by the thermal camera at various distances (four technical replicates at each point). Data are presented as means ± SEM and analyzed using one-way analysis of variance followed by Dunnett's multiple comparison test with a single pooled variance (* $p < 0.05$ significantly different from 10 cm). **(c)** BAT temperatures and the maximum cervix surface temperatures of anesthetized subordinates were simultaneously recorded by a thermoprobe and thermal camera, respectively, every 1 min during the increase in body temperature, following the injection of 1 mg/kg noradrenaline ($n = 3$ animals). Simple linear regression analysis was performed. R^2; coefficient determination.

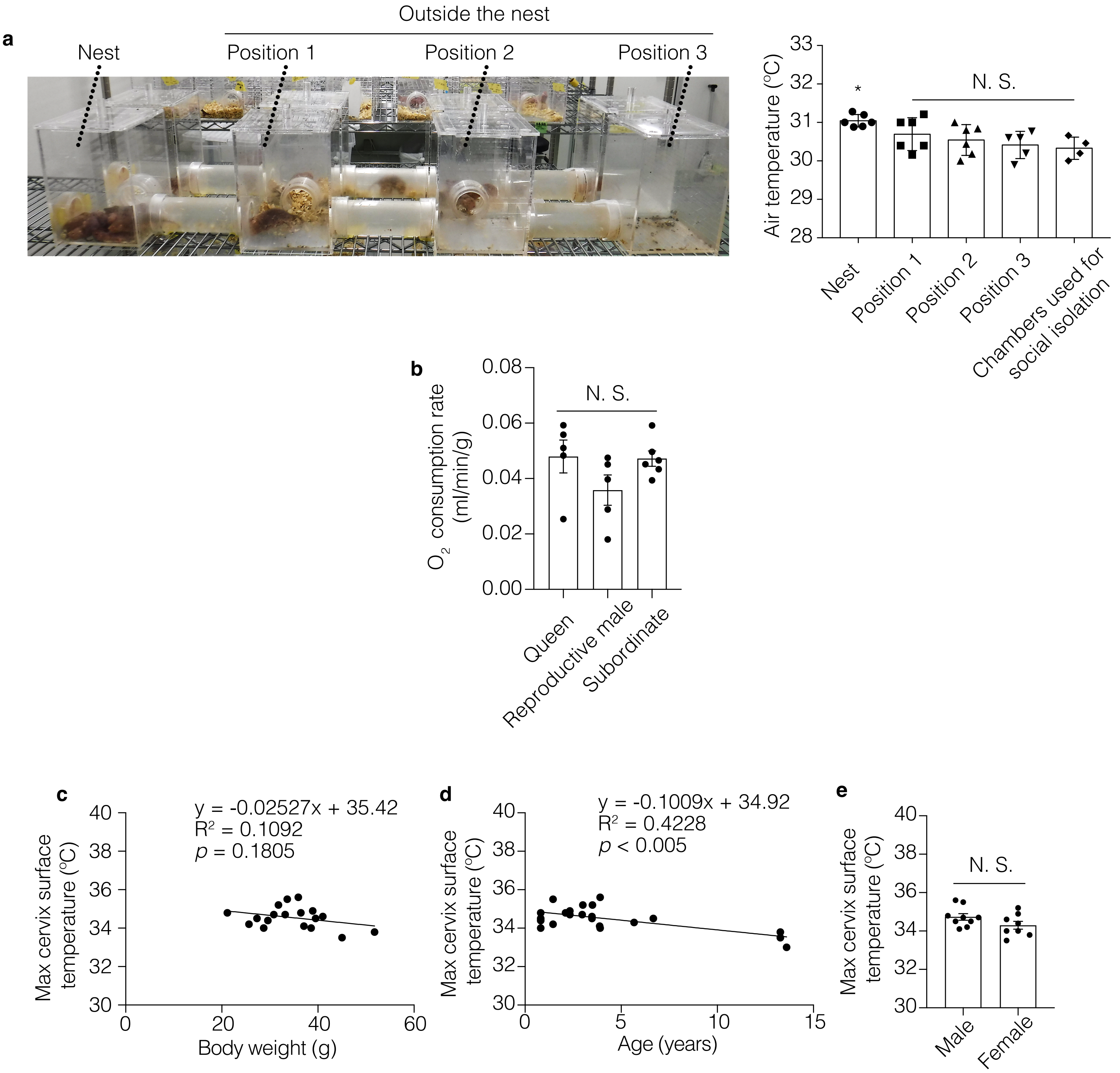
a
Outside the nest
Nest
Position 1
Position 2
Position 3
Air temperature (ºC)
33
32
31
30
29
28
*
N. S.
Nest
Position 1
Position 2
Position 3
Chambers used for social isolation
b
O2 consumption rate (ml/min/g)
0.08
0.06
0.04
0.02
0.00
N. S.
Queen
Reproductive male
Subordinate
c
y = -0.02527x + 35.42
R² = 0.1092
p = 0.1805
Max cervix surface temperature (ºC)
Body weight (g)
0
20
40
60
d
y = -0.1009x + 34.92
R² = 0.4228
p < 0.005
Age (years)
0
5
10
15
e
N. S.
Male
Female

Figure S5. Oxygen consumption rate and relationships between the body temperature and body weight, age, and sex *of* socially isolated subordinate naked mole-rats (NMRs; *Heterocephalus glaber*)

(a) Images and air temperatures (measured using a mercury thermometer) of the colony inside or outside the nest (position 1–3) and inside the chamber used for social isolation. The data are presented as mean ± S.D. and were analyzed using one-way analysis of variance, followed by Dunnett's multiple comparison test with a single pooled variance (the chamber for social isolation was used as a control). * $p < 0.05$. **(b)** Average oxygen consumption rates of the NMRs in a socially isolated state measured in a metabolic cage in a non-cold environment ($n = 5$ queens, 7 subordinates, and 5 reproductive males). Data collected over 4 h in the daytime after a 2-h habituation period. Data are presented as mean ± SEM and were analyzed using one-way analysis of variance, followed by Tukey's multiple comparison test with a single pooled variance for multiple comparisons (N.S. indicates nonsignificant difference between the groups), **(c–e)** Maximum cervix surface temperatures of socially isolated subordinate NMRs monitored by thermal camera according to their **(c)** body weight, **(d)** age, and **(e)** sex.

Simple linear regression analysis was performed in (c) and (d). R^2; coefficient determination. In (e), data are presented as means ± SD. N.S. indicates nonsignificant difference (unpaired *t*-test).

Table S1. Primer sequences

NMR-UCP1-F	CCTCTCCAGTGGATGTGGTG
NMR-UCP1-R	TGCTCAAAGCACACAAACAT
NMR-ZIC1-F	AGCCCTTCAAGTGCGAGTTCGAG
NMR-ZIC1-R	CTTGCAGAGATAGGGCTTGTCAC
NMR-PGC1α-F	CACAGGATCAGAACAAACCC
NMR-PGC1α-R	CAGATACTTGAGAAGCTCCGA
NMR-DIO2-F	AGCTTTCTGCTCGATGCC
NMR-DIO2-R	TCCTGGACACCGTTTCCGCTA
NMR-TBX1-F	ACGGCCACATTATTCTCAACTCCA
NMR-TBX1-R	AAGCGCGTCTCCTCGAACACA
NMR-CITED1-F	CCGGCCCTTCGCTTTCACAC
NMR-CITED1-R	TCAGCTCAGTGGTGCCCCTT
NMR-ACTB-F	AGACCTTCAACACCCCAGCCATGT
NMR-ACTB-R	GGCCAGCCAGGTCCAGACGCAG

Table S2. Relative humidity in our experiments

Location	Relative Humidity
Colony cage at 30°C	69–80%
Isolation cage at 30°C	61–62%
Isolation cage at 20°C	45–50%

Table S3. Giving birth date and experimental date

Experiment	Individual No.	Duration from last giving birth date to experimental date	Duration from experimental date to next giving birth date
Measurement of O_2 consumption rate	1	237	no giving birth
	2	328	130
	3	60	22
	4	47	175
	5	94	132
Monitoring the body surface temperature at 30 °C with Saline	1	118	no giving birth
	2	340	118
	3	118	no giving birth
	4	290	71
	6	414	343
Monitoring the body surface temperature at 30 °C with SR59230A	1	132	no giving birth
	2	354	104
	3	115	no giving birth
	4	287	74
	6	411	346

Video S1. Uptake of 2-deoxy-2-[18F]fluoro-D-glucose ([^{18}F]FDG) in the brown adipose tissue (BAT) of naked mole-rats (NMRs; *Heterocephalus glaber*)

The NMRs were administered 1 mg/kg noradrenaline, after which 11 MBq [^{18}F]FDG was injected into the abdominal cavity. Positron emission tomography/computed tomography (PET-CT) images were then acquired 1 h after [^{18}F]FDG administration. The oblong signal is a microchip for individual identification.
